# The effect of sports shoe design on lower limb function in a neutral foot type

**DOI:** 10.1186/1757-1146-4-S1-P8

**Published:** 2011-05-20

**Authors:** Vivienne Chuter, Richard Smith

**Affiliations:** 1 Podiatry, University of Newcastle, Ourimbah, NSW, 2258, Australia; 2 The University of Sydney, Lidcombe, NSW, Postcode, UK

## Background

Generalised excessive or prolonged foot pronation has been implemented in numerous functional changes to the lower limb resulting in overuse injuries affecting the lower back hip, knee, lower leg, ankle and foot. Motion control components incorporated in midsole of shoes have been based on biomechanical reasoning which assumes excessive motion can be controlled via mechanisms of restraint however this has not be clearly demonstrated in the literature. The aim of this project was to determine the effect of dual density midsoles and neutral midsoles compared with a barefoot condition on the rearfoot kinematics and kinetics in walking gait.

## Methods

Sixteen participants with a Foot Posture Index of 0 to +5 indicating a normal foot type were recruited for this study. Each participant performed walking trials in barefoot, neutral shoe and dual density shoe conditions. A nine-camera, three -dimensional motion analysis system was used to measure frontal plane rearfoot motion and tibial rotation for each condition during the stance phase of gait. Frontal plane rearfoot moments were calculated for each condition.

## Results

There was no significant difference in peak rearfoot frontal plane motion or peak tibial rotation between the barefoot and shoes conditions. The dual density shoe demonstrated a trend towards reduced maximum eversion compared with the barefoot condition (p=0.06). There was a significant increase in peak inversion moment between the barefoot condition and the dual density shoe in walking and running gait (p<0.05). No significant difference in peak rearfoot eversion or peak inversion moment was found between footwear conditions however the neutral shoe was associated with a non-significant increase in peak inversion moment. Time series data demonstrated earlier onset and longer duration of inversion moment in both walking and running gait in the barefoot condition.

## Conclusion

The results of this study indicate that dual density and neutral sports shoes do not have a statistically significant effect on kinematics of the tibia or rearfoot, however there is evidence to suggest a dual density shoe may reduce peak rearfoot eversion. The reduction in inversion moment associated with both types of footwear suggests there may be less demand on anti-pronatory muscles associated with footwear use.

